# 

**Published:** 1910-11-12

**Authors:** 


### THE HOSPITAL

Telegraphic Address: Ospedale, London.

THE MODERN MEDICAL AND INSTITUTIONAL JOURNAL.

Telephone Number: 2734 Gerrard.

NEW SERIES. No. 196, Vol. VIII.

No. 1268, Vol. XLIX.

SATURDAY, NOV. 12TH, 1910.

PRICE THREEPENCE

